# Supplementary material for: The genome sequence of the Clay Triple-lines, Cyclophora linearia (Hübner, 1799) (Lepidoptera: Geometridae)
Source: Wellcome Open Res. 2025 Dec 19;10:679. [Version 1] doi: 10.12688/wellcomeopenres.25389.1 (PMC12961284; doi:10.12688/wellcomeopenres.25389.1)
Supplement: Peer review history

# Reviewer report: Reviewer response for version 1

**DOI:** 10.21956/wellcomeopenres.27973.r142866

**Published:** 2026-03-04

**Authors:** Annabel Whibley

**Affiliations:** 1 Bragato Research Institute, Clyde, New Zealand

Sivess and colleagues report the genome sequence of the Clay Triple-lines, *Cyclophora linearia. *The report follows Darwin Tree of Life pipelines and templates, resulting in a high quality assembly of the two haplotypes, one scaffolded to chromosome level, and presented in comprehensive report of assembly properties,evaluation metrics, sample and sequencing metadata.

 Sample identification was verified by barcoding. Two specimens were used for analysis: ilCycLine1 for the assembly backbone and ilCycLine2 for RNA and HiC. I assume that this sentence “Following whole genome sequence generation, the relevant DNA barcode region was also used alongside the initial barcoding data for sample tracking at the WSI” means that the identify of the second sample was confirmed via checking of the barcode sequence in the new data tranches, but perhaps the verification of the identity of additional specimens could be made more explicit.

 By virtue of having no high coverage contigs, the blobplot is quite compressed towards the top of the y-axis. Perhaps it could be manually rescaled to improve the visualisation?

Are sufficient details of methods and materials provided to allow replication by others?

Yes

Is the rationale for creating the dataset(s) clearly described?

Yes

Are the datasets clearly presented in a useable and accessible format?

Yes

Are the protocols appropriate and is the work technically sound?

Yes

Reviewer Expertise:

Bioinformatics, Genomics

I confirm that I have read this submission and believe that I have an appropriate level of expertise to confirm that it is of an acceptable scientific standard.

# Reviewer report: Reviewer response for version 1

**DOI:** 10.21956/wellcomeopenres.27973.r143294

**Published:** 2026-01-28

**Authors:** Mohadeseh Tahami

**Affiliations:** 1 University of Jyväskylä, Jyväskylä, Finland

The manuscript presents a high-quality, chromosome-scale genome assembly for *Cyclophora linearia*, generated using state-of-the-art PacBio, Hi-C, and RNA-seq approaches. The methods are described very well in details, ensuring reproducibility. The sequencing and assembly statistics are provided in details; the genome assembly includes two haplotypes with total lengths of 280.22 Mb (haplotype 1) and 284.23 Mb (haplotype 2), assembly completeness by BUSCO is estimated 98%, 31 chromosomes were scaffolded including the sex chromosome, and the complete mitochondrial genome is assembled with 16.54 kb length. Biological species background are well described with the rationale of the genome’s value for ecological and evolutionary studies. Minor improvements could include a concise comparative context with related Geometridae genomes. Overall, this is a solid and valuable genome report suitable for indexing.

Are sufficient details of methods and materials provided to allow replication by others?

Yes

Is the rationale for creating the dataset(s) clearly described?

Yes

Are the datasets clearly presented in a useable and accessible format?

Yes

Are the protocols appropriate and is the work technically sound?

Yes

Reviewer Expertise:

evolutionary genomics

I confirm that I have read this submission and believe that I have an appropriate level of expertise to confirm that it is of an acceptable scientific standard.
